# GABA System in Schizophrenia and Mood Disorders: A Mini Review on Third-Generation Imaging Studies

**DOI:** 10.3389/fpsyt.2016.00061

**Published:** 2016-04-19

**Authors:** Chiara Chiapponi, Federica Piras, Fabrizio Piras, Carlo Caltagirone, Gianfranco Spalletta

**Affiliations:** ^1^Neuropsychiatry Laboratory, IRCCS Santa Lucia Foundation, Rome, Italy; ^2^Department of Systems Medicine, University of Rome Tor Vergata, Rome, Italy; ^3^Museo Storico della Fisica e Centro Studi e Ricerche Enrico Fermi, Rome, Italy; ^4^Menninger Department of Psychiatry and Behavioral Sciences, Beth K. and Stuart C. Yudofsky Division of Neuropsychiatry, Baylor College of Medicine, Houston, TX, USA

**Keywords:** GABA, MRS, multimodal imaging, schizophrenia, bipolar disorder, major depressive disorder

## Abstract

Third-generation neuroimaging research has been enriched by advances in magnetic resonance spectroscopy (MRS) measuring the concentration of important neurotrasmitters, such as the inhibitory amino acid GABA. Here, we performed a systematic mini-review on brain MRS studies measuring GABA concentration in patients affected by schizophrenia (SZ), bipolar disorder (BD), and major depressive disorder (MDD). We wondered whether multimodal investigations could overcome intrinsic technical limits of MRS giving a broader view of mental disorders pathogenesis. In SZ, unimodal studies gave mixed results, as increased, decreased, or unaltered GABA levels were reported depending on region, disease phase, and treatment. Conversely, multimodal results showed reduced level of glutamate, but not of GABA, in patients mirrored by *in vitro* biochemical findings revealing hippocampal reduction in glutamate signaling in SZ, and no deficits in GABA synthesis. Moreover, a mouse model confirmed the unique pathological characteristic of glutamate function in SZ. Unimodal studies in BD revealed again, inconsistent results, while no multimodal investigations including MRS on GABA exist. In MDD, unimodal studies could not differentiate patients from controls nor characterize high-risk subjects and remitted patients. However, a multimodal study combining functional magnetic resonance imaging and MRS revealed that cingulate cortex activity is related to glutamate, *N*-acetylaspartate levels and anhedonia in patients, and to GABA concentration in healthy subjects, improving the distinction between MDD and physiology. Overall, our results show that unimodal studies do not indicate GABA as a biomarker for the psychiatric disorders considered. Conversely, multimodal studies can widen the understanding of the link between psychopathology, genetics, neuroanatomy, and functional–biochemical brain activity in mental disorders. Although scarce, multimodal approaches seem promising for moving from GABA MRS unimodal-descriptive to causal level, and for integrating GABA results into a more comprehensive interpretation of mental disorder pathophysiology.

## Introduction

An imbalance between excitation and inhibition in brain neuronal transmission has been hypothesized as one of the molecular mechanisms responsible for psychiatric disorders (1–5). In this context, multimodal studies coupling the continuous technical progresses in neuroimaging to methods for measuring neurotramsitter concentrations may represent a turning point for *in vivo* evidence of postmortem (6–8) and animal model (9–12) results. Moreover, the chance to link psychopathology, genetics, neuroanatomy, and functional–biochemical brain activity may take psychiatric research to the causal understanding of patients’ illness.

The support given by newly developed improvements in well known technologies, such as proton magnetic resonance spectroscopy (MRS) (13–15), has been fundamental to encourage *in vivo* research on gamma-aminobutyric acid (GABA) in brain physiology and pathology (16–18). GABA is the primary inhibitory neurotransmitter in the mammalian central nervous system. Theories on its dysfunction in schizophrenia (SZ) assume that alterations in the neural circuitry involving GABA have a role in the mechanisms of the disorder and associated cognitive deficits (19–21). The role of GABA dysfunction in different psychiatric disorders such as bipolar disorder (BD), or major depressive disorder (MDD) is also established (3, 22, 23).

Magnetic resonance spectroscopy is the election technique to non-invasively measure *in vivo* GABA concentration in selected brain regions (18, 24). However, direct interpretation of MRS results is limited by intrinsic features of the technique. In particular, acquisition of GABA signal is restricted to large (e.g., 3×3×3 cm^3^) single voxels, since multi-voxel spectroscopy usually measures metabolites with longer T2 relaxation, such as *N*-acetylaspartate (NAA), choline (Cho), and creatine (Cr). This results in a broad between-studies heterogeneity in the anatomical region investigated. Moreover, MRS can only detect total concentration of neurochemicals and cannot distinguish between separate functional pools, thus impeding conclusions on neurotransmitters availability.

In this context, multimodal approaches, combining MRS with other complementary techniques, would lead to a solid and comprehensive interpretation of neurochemical underpinnings of brain pathologies. As a case in point, multimodal MRS and functional magnetic resonance imaging (fMRI) would help in depicting the neurochemical and functional pathological mechanisms responsible for complex disorders. The support from electrobiological measurements such as electroencephalography (EEG) or magnetoencephalography (MEG), measuring the oscillatory activity in brain neuronal ensembles, could be fundamental in interpreting results on GABA concentration since the latter has been shown to be positively correlated with stimulus specific neuronal oscillations (25–27). Similarly, findings from *in vitro* tissue biochemistry, animal models, and genetics could provide data at higher spatial resolution and further mechanistic insights into the interpretation of GABA concentration (28).

On the basis of these considerations, we reviewed research articles focusing on GABA as measured by MRS in SZ and mood disorders (i.e., BD and MDD). In particular, we analyzed whether studies combining different approaches could overcome the technical limits intrinsic to MRS and give a broader view of the mechanisms involved into mental disorders.

## Methods

To investigate recent MRS studies evaluating GABA level in the brain, we performed a systematic literature search on PubMed, PsycNET (including PsycINFO, PsycBOOKS, PsycCRITIQUES, PsycARTICLES, and PsycEXTRA databases), and Scopus database till November 2015 using the keywords “GABA” *AND* “spectroscopy” *AND* any of the following terms: “schizophrenia,” “bipolar disorder,” “major depressive disorder.” The reference list of identified articles and review papers was also hand searched to obtain additional articles. Inclusion criteria for studies selection were (1) English language, (2) articles published in peer-reviewed journals after 2000, (3) original research article (comments, letters to editors and review articles were excluded), (4a) inclusion of patients diagnosed with the specific neuropsychiatric disorder of interest according to ICD or DSM criteria or (4b) inclusion of high risk (HR) subjects, (5) inclusion of at least 10 patients, (6) comparison between patients and healthy controls (HC), (7) performance of MRS using a magnetic field of at least 3 T (to have a good signal-to-noise ratio and to resolve GABA peak from those of other more concentrated molecular compounds, e.g., NAA or Cr).

In the search for SZ studies, 72 papers were initially identified. Among them, 11 were not original researches (9 reviews, 1 comment, and 1 letter), 2 studies did not consider HC and 9 did not include SZ patients, 22 papers did not include humans (e.g., studies on animal models and *in vitro* measurements), 9 studies measured the unresolved glutamate + glutamine (Glx) with GABA contamination peak as a proxy of GABA concentration, 1 study included less than 10 patients and 6 studies were published before 2000. At the end of the selection process, 12 studies on SZ fulfilled the inclusion criteria.

In the search for BD studies, 21 papers were screened, but we excluded 7 reviews, 3 studies not performing *in vivo* MRS on humans, 1 on healthy men only, 1 not measuring GABA, 3 studies considering Glx, and 1 including less than10 patients. Only five studies survived the selection process for BD.

At last, 53 studies were initially identified for MDD, but only 11 studies were eligible for the review, and 42 were excluded (6 studies without a control group, 5 not focusing on MDD patients, 6 not using *in vivo* MRS on humans, 11 reviews, 1 comment, 5 measuring Glx, 4 considering less than 10 patients, 3 not in English, and 1 published before 2000).

## Results

### Schizophrenia

GABA MRS results in SZ are very scattered, since GABA concentration was found reduced, increased, or unaltered in patients (see Table 1). Such heterogeneity is mostly due to the different methodological approaches used, as studies vary in terms of patients’ clinical characteristics, brain region under investigation, and aims of the studies. Indeed, while most authors evaluated the diagnosis effect on GABA concentration, others considered the effect of age, of antipsychotics, and the role of GABA in different illness phases.

**Table 1:** **Studies comparing GABA concentration between SZ patients and HC**.

Reference	Sociodemographic characteristics				Clinical characteristics			Probed brain region	Brain region of altered GABA in patients	Additional findings	Other techniques
	Sample size		Age [mean (SD) or years range]		Illness duration [mean (SD) or years range]	GMM [no. patients (%)]	Antipsychotics [no. patients (%)]				
	
	Patients	HC	Patients	HC
**SZ < HC**											

Rowland et al. (30)	31 older	37 older	48.3 (5.8)	51.0 (6.0)	24.0 (9.8)	Anticholinergics: 1 (3)	Typ: 4 (13); Atyp: 18 (58); Both: 6 (19); none: 3 (10)	MFC	MFC		
	29 younger	40 younger	25.7 (4.3)	25.3 (4.6)	5.6 (4.6)	Anticholinergics: 2 (7)	Typ: 1 (3); Atyp: 25 (87); Both: 1 (3); None: 2 (7)	MFC			
Rowland et al. (29)	11 younger	10 younger	30.2 (6.6)	33.4 (6.5)	7.7 (4.1)	Benzodiazepines or mood stabilizers free at scan time	Atyp: 11 (100)	ACC, CSO	ACC		
	10 older	10 older	51.1 (4.0)	49.4 (3.9)	25.5 (6.5)	Benzodiazepines or mood stabilizers free at scan time	Typ: 2 (20); Atyp: 8 (80)	ACC, CSO			
Marsman et al. (31)	17	23	27.6 (6.1)	27.7 (5.3)	6.4 (6.8)	Benzodiazepines current: 6 (35) Benzodiazepines lifetime: 11 (65)	Typ: 3 (18); Atyp: 10 (59); Both: 4 (23)	PFC, POC	PFC	GABA reduction independent from antipsychotics dosage and benzodiazepines use	
Kelemen et al. (32)	28	20	24.9 (8.3)	24.2 (6.9)	<1	T0: drug naive: 28 (100); FU: anticholinergics: 5 (18); benzodiazepines: 16 (57); mood stabilizers: 5 (18)	T0: None: 28 (100); FU: Typ: 3 (11); Atyp: 25 (89)	OC	OC	GABA_SZ_T0 = GABA_SZ_FU	
Yoon et al. (33)	13	13	27.5 (8.8)	28.1 (8.2)	Na	Na	Typ: 1 (8); Atyp: 7 (54); None: 5 (38)	OC	OC	Medication dosage did not influence results	
Goto et al. (34)	16	18	30 (11)	15–49	<0.5	Na	T0: None: 16 (100); FU: Atyp: 16 (100)	MFC, ltBG, POC	ltBG	GABA_SZ_T0 = GABA_SZ_FU	

**SZ = HC**											

Stan et al. (28)	18	16	41.94 (8.5)	35.63 (11.74)	Na	Anticonvulsants: 1 (5); benzodiazepines: 2 (11), valproic acid: 2 (11)	Typ, Atyp, Both: Na; None: 7 (39)	Hippocampus		Hippocampal GABA *in vivo*, *in vitro* and in animals did not differ between SZ and controls	Hippocampal dissection and tissue immunoblotting on postmortem SZ patients
											Animal MRS on the DG-selective GRIN1 knockout mice
Chen et al. (36)	12	12	31.00 (10.79)	33.08 (8.23)	Na	Na	Atyp: 9 (75); None: 3 (25)	DLPFC		Correlation between GABA levels and gamma band oscillation in SZ and HC	EEG at rest EEG during a working memory task
Tayoshi et al. (35)	38	29	34.0 (10.0)	34.0 (10.2)	11.1 (9.4)	Benzodiazepines: 16 (42)	Typ: 16 (42); Atyp: 22 (58)	ACC, ltBG		ltBG: GABA_Typ > GABA_Atyp	

**SZ > HC**											

De la Fuente-Sandoval et al. (39)	23 UHR	24	20.7 (4.1)	21.4 (3.3)	<1	Medication free for T >12 weeks: 23 (100)	None: 23 (100)	Dorsal caudate, MPFC	Dorsal caudate, MPFC		
Kegeles et al. (37)	16 unmed^a^, 16 med	22	unmed: 32 (11), med: 32 (10)	33 (8)	unmed: 7 (7), med: 9 (8)	Benzodiazepines free at scan time: 32 (100)	med: Atyp: 16 (100)	MPFC, DLPFC	MPFC	GABA_SZ_unmed > GABA_HC GABA_SZ_med = GABA_HC
Ongür et al. (38)	21	19	39.0 (10.8)	36.3 (9.8)	Na	Anticonvulsants: 6 (28); benzodiazepines: 10 (48); lithium: 4 (19)	Typ: 3 (14); Atyp: 16 (76); Both: 1 (5); None: 1 (5)	ACC, POC	ACC + POC, averaged		

*^a^Patients free of antipsychotic medication treatment for a minimum of 14 days prior to the scan*.

*ACC, anterior cingulate cortex; Atyp, patients taking atypical antipsychotics; CSO, centrum semiovale region; DG, dentate gyrus; DLPFC, dorsolateral prefrontal cortex; EEG, electroencephalography; FU, follow up; GMM, GABA-modulating medication; HC, healthy controls; ltBG, left basal ganglia; med, medicated; MFC, medial frontal cortex; MPFC, medial prefrontal cortex; Na, not available; OC, occipital cortex; PFC, prefrontal cortex; POC, parieto-occipital cortex; SD, standard deviation; SZ, schizophrenia patients; T, time; T0, baseline; Typ, patients taking typical antipsychotics; UHR, ultra high risk patients; unmed, unmedicated*.

The most reported result (i.e., replicated in six studies) is that GABA concentration is reduced in SZ patients with respect to HC (29–34). Specifically, GABA was reduced in medial frontal cortex (MFC) (29, 30) and occipital cortex (OC) (32, 33), and the result was modulated by age in MFC (29, 30) and not affected by current medication type or dosage in OC (32, 33). The observed reduction in MFC GABA level in old SZ subjects as compared to age-matched HC suggests that GABA concentration decreases as age increases in patients and not in controls (29, 30). The independence from medication dosage in the OC (33) was further extended to the basal ganglia (34) suggesting that GABA reduction in these areas is driven by the disorder, being observable also in first-episode patients (32), and not an effect of treatment. A reduced GABA level in prefrontal areas of SZ patients was described only performing MRS at very high (7 T) magnetic field (31). Conversely, three studies (34–36) failed to find alterations in GABA level in SZ with respect to HC in any of the considered regions. Among them, one study found that patients taking only typical antipsychotics had higher GABA concentration than those taking only atypical antipsychotics (35). The other two studies failing to find GABA alterations in SZ, probed the hippocampus and the dorsolateral prefrontal cortex (DLPFC) (28, 36). These studies are of particular interest since they combined MRS with different experimental techniques. In particular, one correlated GABA levels in DLPFC to gamma oscillations, as measured by EEG during a working memory task, and found that both baseline and working memory-induced gamma oscillations were strongly dependent on GABA levels either in patients and controls (36). Within a data rich experimental design, the second multimodal study integrated *in vivo* MRS measurements of hippocampal GABA (and glutamate) concentration in patients with *in vitro* tissue biochemistry (sampling postmortem human hippocampal tissue) and MRS on a mouse model recapitulating symptoms of SZ (dentate gyrus-selective knockout of the GRIN1 gene, encoding a critical unit of *N*-methyl-d-aspartate receptors) (28). Looking at *in vivo* MRS results, authors found no global difference in GABA level between SZ and HC both in humans and animals, while they found decreased glutamate in SZ. Looking at *in vitro* results, authors found reduced level of GluN1 protein, a marker of the glutamatergic system, in SZ, but no alterations with respect to HC in the level of GAD67, the main enzyme in the GABAergic system. The combination of such findings provides evidence that the excitatory, but not the inhibitory, system within the hippocampus is implicated in SZ pathogenesis.

Finally, three studies found increased GABA concentration in SZ with respect to HC. One of them compared unmedicated SZ and patients medicated only with atypical antipsychotics to HC (37). Authors showed increased prefrontal GABA in unmedicated SZ patients with respect to both medicated and HC samples. Such results partially confirmed those presented in a previous research in which, averaging GABA concentration in anterior cingulate cortex (ACC) and parieto-occipital cortex (POC), authors found increased GABA in chronic SZ (38). More recently, an increased GABA concentration in dorsal caudate area and medial prefrontal cortex has been observed also considering ultra HR patients free from GABA modulating medications (GMM) (i.e., benzodiazepines, mood stabilizers, or antidepressants) and antipsychotics (39).

### Bipolar Disorder

Among the few studies using MRS to measure GABA concentration in BD, three reported no difference between patients and HC (40–42). However, papers contributing to such evidence are very heterogeneous in terms of localization of MRS voxel, clinical characteristics of BD samples, and GMM (see Table 2), which were scarcely considered in the analyses. Their effect was specifically taken into account in a study indicating an increased GABA in BD as a whole, with respect to HC. However, within the patients group, there was a reduction of GABA in those taking GMM (43). To clarify the impact of medication dosage and lifetime exposure on GABA concentration, some authors considered only drug free patients (for at least 3 months before MR scan) who however, had lifetime exposure to lithium, antidepressants, or mood stabilizers (44). Results indicated decreased GABA level in recovered unmedicated BD patients with respect to HC.

**Table 2:** **Studies comparing GABA concentration between mood disorders patients (BD and MDD) and HC**.

Reference	Sociodemographic characteristics				Clinical characteristics			Probed brain region	Brain region of altered GABA in patients	Additional findings	Other techniques
	Sample size		Age [mean (SD) or years range]		Illness duration (mean (SD) or years range)	GMM [no. patients (%)]	Antipsychotics [no. patients (%)]				
	
	Patients	HC	Patients	HC
**BD**											

***BD*** **<** ***HC***											
Bhagwagar et al. (44)	16 BD-I, 15 rMDD	18	BD-I = 37.0 (13.8), rMDD = 42.1 (14.6)	37.6 (14)	BD-I = 0.5–10.1, rMDD = 1–18.4	Medication free for *T* ≥ 3 months: 32 (100), Lifetime exposure: BD-I: antidepressant: 11 (69); lithium: 6 (37); mood stabilizers: 3 (19). rMDD: antidepressant: 10 (62); lithium: 1 (6)	Na	OC	OC	GABA_rMDD = GABA_BD-I	
***BD*** **=** ***HC***											
Soeiro-de-Souza et al. (40)	50	38	31.7 (9.1)	25.7 (5.7)	Na	anticonvulsants: 23 (46); antidepressants: 8 (16); benzodiazepines: 1 (2); lithium: 29 (58)	Atyp: 23 (46), Typ, Both: Na; None: 0 (0)	ACC			
Godlewska et al. (41)	13	11	23.8 (3.6)	21.9 (2.7)	Na	Mood stabilizers naive: 13 (100)	None: 13 (100)	MPFC, OC			
Kaufman et al. (42)	13	11	40.5 (12.5)	41.2 (14.0)	18.4 (11.4)	Antidepressant: 6 (46), mood stabilizers: 12 (92)	Typ, Atyp, Both: Na; None: 0 (0)	POC, Thal, whole brain		Whole brain: GABA_BD_antipsy < GABA_BD_noantipsy	
***BD*** **>** ***HC***											
Brady et al. (43)	14 BD-I	14	32.6 (13.6)	36.9 (10.4)	8.7	Anticonvulsants: 5 (36); antidepressants: 7 (50); benzodiazepines: 6 (43); lithium: 4 (29)	Typ: 2 (14); Atyp: 9 (64); Both: Na; None: 0 (0)	ACC, POC	ACC, POC	GABA_HC < GABA_BD-I_GMM < GABA_BD-I_nGMM	

**MDD**											

***MDD*** **=** ***HC***											
*High risk patients*											
Taylor et al. (45)	24 HR	28	18.9 (16–21)	19.8 (17–21)	0	Drug naive: 24 (100)	None: 24 (100)	POC			
*Patients depressed at scan time*											
Godlewska et al. (46)	39	31	29.9 (10.6)	30.3 (10.6)	Na	6 weeks FU: antidepressant (escitalopram): 39 (100)	T0: None: 39 (100)	OC		T0: GABA_MDD = GABA_HC GABA_MDD_T0 = GABA_MDD_FU	
Preuss et al. (47)	19 cMDD, 16 rMDD, 9 PD	20	cMDD: 31.5 (9), rMDD: 40.8 (11.7), PD = 33.8 (12.8)	36.9 (13.8)	Na	Psychotropic medication free for *T* > 4 weeks: 44 (100)	None	PFC		GABA level differs between female carrier/non-carrier of 3 nuclear polymorphysms	Genotyping
Walter et al. (48)	19 (11 with MRS GABA level)	24 (13 with MRS GABA level)	40.0 (Na)	34.6 (Na)	Na	Psychotropic medication free for *T* > 1 week: 19 (100)	Na	ACC		GABA_HC correlated with NBR, but not GABA_MDD	fMRI
*Patients remitted at scan time*											
Shaw et al. (49)	19	18	23 (2.6)	21 (1.5)	Na	Medication free: 19 (100)	Na	PFC, OC, ltBG		OC: IGF_rMDD = IGF_HC	MEG
Hasler et al. (50)	16	15	41.0 (11.6)	41.7 (12.4)	Na	Antidepressant medication free for *T* ≥ 3 months: 16 (100)	Na	DM/DA-PF, VM-PF			
***MDD*** **<** ***HC***											
*Patients depressed at scan time*											
Gabbay et al. (51)	20	21	16.7 (2.7)	16.2 (1.6)	11.7 (8.6) months	Psychotropic medication free for *T* ≥ 3 months: 20 (100)	Na	ACC	ACC		
Price et al. (52)	15 TRD, 18 nTRD	24	TRD = 46.8 (11.9), nTRD = 38.3 (12.3)	37.25 (13.5)	TRD: 26.93 (10.8), nTRD: 21.80 (16.4)	Psychotropic medication-free for *T* ≥ 2 weeks: TRD + nTRD: 33 (100)	Na	OC, ACC	OC	OC: GABA_MDD (TRD + nTRD) < GABA_HC	
										GABA_TRD < GABA_HC GABA_nTRD = GABA_HC	
Hasler et al. (53)	20	20	34.0 (11.2)	34.8 (12.4)	18.8 (13.5)	Medication free for *T* > 4 weeks or medication naive: 20 (100)	Na	DM/DA-PF, VM-PF	DM/DA-PF		
*Patients remitted at scan time*											
Bhagwagar et al. (54)	12	11	40.6 (4.2)	34.3 (4.1)	Na	Medication free for *T* > 6 months: 12 (100)	Na	ACC, POC	ACC, POC		
Bhagwagar et al. (44)	16 BD-I, 15 rMDD	18	BD = 37.0 (13.8), rMDD = 42.1 (14.6)	37.6 (14)	BD-I: 0.5–10.1, rMDD: 1–18.4	Medication free for *T* ≥ 3 months: 32 (100), Lifetime exposure: BD-I: antidepressant: 11 (69); lithium: 6 (37); mood stabilizers: 3 (19). rMDD: antidepressant: 10 (62); lithium: 1 (6)	Na	OC	OC	GABA_ rMDD = GABA_BD-I	

*ACC, anterior cingulate cortex; Atyp, patients taking atypical antipsychotics; BD, bipolar disorder patients; BD_antipsy, patients taking antipsychotics; BD-I_GMM, BD-I patients taking GABA modulating medications; BD-I, patients with bipolar disorder type I; BD_noantipsy, patients not taking antipsychotics; BD-I_nGMM, BD-I patients not taking GABA modulating medications; cMDD, patients with a current episode of major depressive disorder; DM/DA-PF, dorsomedial/dorsal anterolateral prefrontal region; fMRI, functional magnetic resonance imaging; FU, follow up; GMM, GABA-modulating medication; HC, healthy controls; HR, high risk patients; IGF, induced γ frequency; ltBG, left basal ganglia; MEG, magnetoencephalography; MDD, major depressive disorder patients; MPFC, medial prefrontal cortex; Na, not available; NBR, negative blood response; nTRD, non-treatment-resistant depression; OC, occipital cortex; PD, panic disorder; PFC, prefrontal cortex; POC, parieto-occipital cortex; rMDD, individuals with remitted major depressive disorder; SD, standard deviation; T, time; T0, baseline; Thal, thalamic region; TRD, treatment-resistant depression; Typ, patients taking typical antipsychotics; VM-PF, ventromedial prefrontal region*.

No study using multimodal techniques has been published so far on BD patients.

### Unipolar Major Depressive Disorder

Studies investigating unipolar MDD patients showed either no difference in GABA concentration between patients and HC (45–50), either a reduction of GABA in MDD (44, 51–54). A decreased GABA level has been observed mainly in patients depressed at scan time (51–53), but some authors found a reduction also in remitted patients (44, 54). One study comparing GABA level between HR subjects (i.e., having a family history of parental depression) and a control group without a family history of depression described negative results (45). Among studies failing to find an alteration of GABA in MDD, one combined genotyping with MRS in order to test the effect of common variants of the tryptophan hydroxylase isoform 2 (TPH2) gene, modulating serotonergic neurotransmission and brain circuits for emotion and adaptation, on GABA concentration in the prefrontal cortex (PFC) (47). Authors found a significant association between increased GABA concentration in the PFC and the allele frequencies of three TPH2 SNPs in female subjects, independently from diagnosis. Along with MRS, another research focused on remitted, formerly severe MDD patients and HC using MEG to measure the induced gamma oscillation frequency (IGF), a reliable surrogate marker of postsynaptic GABA function, in the OC (49). Authors found that MDD have normal IGF and GABA concentration in the OC. In a further multimodal investigation, MRS quantifying GABA, glutamate, and NAA concentrations was combined with fMRI measuring blood oxygenation level-dependent (BOLD) response to emotional stimuli in the pregenual ACC, part of the default mode network, related to anhedonia (48). MRS results showed no alteration in metabolites concentration in MDD patients, while fMRI indicated that negative BOLD responses, as well as glutamate and *N*-acetylaspartate concentrations, correlated with emotional intensity ratings, an anhedonia surrogate, in MDD but not in HC. Differently, negative BOLD responses in HC correlated with GABA. The fact that GABA concentration could not differentiate between MDD patients and HC together with the absence of GABA modulating effects on anhedonia were interpreted as secondary outcomes consequent to a primary deficit in glutamatergic metabolism, which may lead to a distortion of the excitation–inhibition balance and cause anhedonic depression.

## Discussion

The involvement of GABA abnormalities in the mechanisms of psychiatric disorders is strongly debated. In particular, recent developments in MRS sequences allow discriminating the peak of GABA from those of more concentrated metabolites in the brain, thus permitting its measurement. However, despite postmortem evidence and preclinical studies highlighting GABAergic abnormalities in patients with mental disorders, the connection between these abnormalities and categorical/diagnostic or dimensional/symptomatic characteristics is still unclear. In this framework, we reviewed the body of evidence on GABA concentration, as measured by MRS in localized brain regions of SZ, BD, and MDD patients, particularly highlighting results obtained by multimodal methods and multiple experimental techniques.

Although this topic is under continuous development, some conclusions can be drawn from the present results.

### Schizophrenia

First, the reduction of GABA level in SZ (the most frequent reported result) seems to occur in specific brain areas (frontal, occipital, and basal ganglia) and in old age, being probably a mixed effect of chronicity, lifetime exposure (more than current type or dosage) to antipsychotics, and GMM, particularly benzodiazepines (17). The latter is known to allosterically increase GABA_A_ receptor activation, but available experimental techniques are still too coarse to detect circuit-specific perturbations in GABA levels as induced by benzodiazepines (or other medications modulating neuronal transmission), and results are not concordant. From our review, a slight majority of authors failed to find a link between GABA level and medications. Such heterogeneous results might be reconciled performing technically more precise experiments (e.g., MRS at ultra high magnetic field) and enrolling HR subjects in their preclinical stage or drug naive patients to be followed longitudinally.

The second interesting conclusion derived from multimodal studies on SZ is that GABA concentration alone cannot be considered a biomarker for this disorder, while a potential perturbation in the balance between excitation and inhibition, measurable through glutamate/GABA ratio, needs to be more deeply investigated in SZ (28). The latter should be the target for studies aimed at clarifying mechanisms and/or novel therapeutic strategies.

### Bipolar Disorder

Unfortunately, GABA cannot be considered a biomarker of BD yet. Indeed, the only study including young and drug naive patients failed to find differences with respect to HC (41). From the other few studies, it appears that both current and lifetime exposure to GMM tend to reduce GABA level in BD patients, especially in the OC (43, 44). However, heterogeneity of patients’ clinical characteristics, illness phase at scan time, number of previous manic/depressive episodes, and eventual action of the complex mixtures of GMM (not only benzodiazepines but also antidepressants, lithium, mood stabilizers, etc.) justify the need to start multimodal researches focused on more ­homogeneous clinical subsamples.

### Major Depressive Disorder

Research on neurotransmission in MDD is truly promising and intriguing in the hunt for innovative approaches to prevention. Understanding whether eventual changes in GABA reflect an underlying trait vulnerability to depression, or can be considered “scars” of depressive episodes or treatment effects, may have implications for preventative strategies in HR subjects (55). The only study measuring GABA concentration with MRS in subjects at risk of depression did not find differences in the parieto-occipital cortex with respect to subjects not at risk, indicating that, at the actual level of accuracy, GABA level in such brain region cannot be considered an endophenotype for depression (45). Moreover, the study including genotyping showed that GABA concentration in PFC is associated with allele frequencies of three polymorphisms linked to anxiety only in women, independently from the diagnosis (47). This result reinforces the notion that GABA levels are not a marker of MDD (at least in the POC and PFC). The other two multimodal studies associating MRS with fMRI (48) and MEG (49) failed to find differences in GABA concentration in diffuse brain regions between MDD and HC. However, the classification of studies in terms of patients state (i.e., depressed/remitted) at scan time (see Table 2) allows us to support the idea that GABA level identifies the state of being ill, and is not a trait marker for diagnosis, since physiological concentration has been described in the majority of studies including MDD patients during the remission phase (44, 49, 50, 53, 54). Conversely, a primary deficit in glutamatergic metabolism may cause aberrant neuronal activations patterns in regions specifically relevant for the expression of anhedonic behavior in MDD.

## Conclusion

Complex and multimodal researches looking at GABA in psychiatric populations are still a minority. Our review shows that fMRI, *in vitro* biochemistry, genotyping, EEG, and MEG have been combined to MRS, and each of them adds a piece to the puzzle depicting the role of GABA abnormalities in psychiatric disorders. Indeed, fMRI can differentiate neural response patterns induced by stimulation (56), *in vitro* biochemistry allows higher resolution spatial information and correlations between MRS results and biochemical activity of the brain, while genotyping can elucidate the genetic correlates of GABAergic transmission. Furthermore, as EEG reflects voltage changes resulting from the synchronous firing of groups of neurons (57), and MEG describes the effects of synchronous postsynaptic activity (58), when combined with MRS they allow the *in vivo* investigation of GABA effect on neuronal transmission. Thus, from studies using a multimodal approach, it appears that GABA level alone may not be the best biomarker for the psychiatric disorders here considered. However, it is a promising parameter, particularly for the stratification of patients in more homogeneous subtypes sharing specific biological features. The possibility to reduce heterogeneity in psychiatric patients is fundamental both in research (giving the opportunity to gain new insight in the underlying pathophysiology of different mental disorders) and in clinical practice (allowing the prescription of effective and tailored medical treatments).

Conversely, although still scarce, the so-called third-generation paradigms will be the turning point of neuroimaging research on neurotransmission in general, and on GABA dysfunctions in particular. The effort spent in the design and realization of multimodal studies, as well as multicentre ones to include larger samples, would then be rewarded by the strong translational impact of such researches. This approach would support clinicians in the design of preventative interventions with defined, expected outcomes for specific types of psychiatric patients making “precision medicine” a more realistic medical model. The precise medicine is the final end.

## Author Contributions

CCh and GS conceived the paper and performed literature search. CCh, FeP, FaP, and GS wrote the paper. All authors critically reviewed the manuscript and agreed on its final version.

## Conflict of Interest Statement

The authors declare that the research was conducted in the absence of any commercial or financial relationships that could be construed as a potential conflict of interest.
